# Professor Hufeland

**Published:** 1836-10

**Authors:** 


At Berlin, August 26th, the celebrated professor and physician, C. W. Hufeland, in the 75th year of his age, after an illness of four weeks. Hufeland was born on the 12th of August, 1762. As a practising physician, a teacher of medicine, and a medical writer, Dr. Hufeland has been universally known during the last forty years, and, we may add, has been as generally respected as known. We shall give some account of his life and writings in a future Number.

